# Host targeted antiviral (HTA): functional inhibitor compounds of scaffold protein RACK1 inhibit herpes simplex virus proliferation

**DOI:** 10.18632/oncotarget.26907

**Published:** 2019-05-14

**Authors:** Hemayet Ullah, Wangheng Hou, Sivanesan Dakshanamurthy, Qiyi Tang

**Affiliations:** ^1^ Department of Biology, Howard University, Washington, DC 20059, USA; ^2^ Department of Microbiology, Howard University College of Medicine, Washington, DC 20059, USA; ^3^ Department of Oncology, Clinical and Experimental Therapeutics Program, Lombardi Comprehensive Cancer Center, Georgetown University Medical Center, Washington, DC 20057, USA

**Keywords:** host targeted antiviral (HTA), herpes simplex virus (HSV), receptor for activated C kinase 1 (RACK1), RACK1 inhibitor, internal ribosomal entry site (IRES)

## Abstract

Due to the small number of molecular targets in viruses and the rapid evolution of viral genes, it is very challenging to develop specific antiviral drugs. Viruses require host factors to translate their transcripts, and targeting the host factor(s) offers a unique opportunity to develop broad antiviral drugs. It is well documented that some viruses utilize a host protein, Receptor for Activated C Kinase 1 (RACK1), to translate their mRNAs using a viral mRNA secondary structure known as the Internal Ribosomal Entry Site (IRES). RACK1 is essential for the translation of many viruses including hepatitis C (HCV), polio, Drosophila C (DCV), Dengue, Cricket Paralysis (CrpV), and vaccinia viruses. In addition, HIV-1 and Herpes Simplex virus (HSV-1) are known to use IRES as well. Therefore, host RACK1 protein is an attractive target for developing broad antiviral drugs. Depletion of the host’s RACK1 will potentially inhibit virus replication. This background study has led us to the development of novel antiviral therapeutics, such as RACK1 inhibitors. By utilizing the crystal structure of the RACK1A protein from the model plant *Arabidopsis* and using a structure based drug design method, dozens of small compounds were identified that could potentially bind to the experimentally determined functional site of the RACK1A protein. The SPR assays showed that the small compounds bound strongly to recombinant RACK1A protein. Here we provide evidence that the drugs show high efficacy in inhibition of HSV-1 proliferation in a HEp-2 cell line. The drug showed similar efficacy as the available anti-herpes drug acyclovir and showed supralinear effect when applied in a combinatorial manner. As an increasing number of viruses are reported to use host RACK1 proteins, and more than 100 diverse animals and plant disease-causing viruses are known to use IRES-based translation, these drugs can be established as host-targeted broad antiviral drugs.

## INTRODUCTION

With the small number of molecular targets in viruses and the rapid evolution of viral genes, it is very challenging to develop specific antiviral drugs. Unlike other infectious agents, viruses offer few intrinsic targets for inhibition by antiviral molecules [1]. With their simple structural form and their ability to hijack molecular machinery from host cells to complete their replication cycle, viruses evade most efforts to contain them [2]. However, as viruses require host factors to translate their transcripts, targeting the host factor(s) offers a unique opportunity to develop novel antiviral drugs. In this regard, identification of the ribosome localized host protein Receptor for Activated C Kinase 1 (RACK1) for viral Internal Ribosomal Entry Site (IRES)-mediated translation of non-capped mRNAs has been established as a target for developing antiviral drugs [3, 4]. Because IRES-utilizing viruses use this unique mechanism for translation, it has been hypothesized that specific inhibitors of IRES-dependent translation could be therapeutic for viral infections [5]. In addition to regulating the cap-independent IRES-mediated translation of some RNA viruses, RACK1 has recently been shown to regulate translation of capped mRNAs encoded by vaccinia virus (VacV), a DNA virus [6]. Pox virus infection leads to the modification of host RACK1 to mimic a plant-like state that remodels the host ribosome, such that Poly-A repeat-containing mRNAs erroneously generated by slippage of the viral RNA polymerase confer a translational advantage to the virus [6].

It is obvious that the use of any kind of internal ribosome entry site should be especially advantageous for single-stranded + RNA viruses lacking the cap structure. Indeed, many of the animal and human viruses containing IRESs are RNA viruses, and most of them belong only to three families of + ssRNA viruses (Picornaviridae, Dicistroviridae and Flaviviridae). To date, although IRES elements have been widely reported in RNA viruses, only a very limited number of IRES elements have been reported in a few DNA viruses, including Kaposi’s sarcoma-associated herpesvirus (KSHV) [7–9], Epstein-Barr virus (EBV) [10], herpes simplex virus (HSV) [11], murine gammaherpesvirus 68 (MHV-68) [12], Marek’s disease virus (MDV) [13], and simian vacuolating virus 40 (SV40) [14]. In addition, presence of the IRES in double stranded DNA containing White Spot Syndrome virus (WSSV), which is in the family Nimaviradae, is also reported [15]. WSSV is widely known as the causative pathogen of a serious disease in shrimp [15]. The IRES website documents more than 68 viruses with references for IRES evidence, including Human immunodeficiency virus type 1(HIV-1), Human immunodeficiency virus type 2 (HIV-2); Herpes Simplex Virus-1 (HSV-1), Epstein-Barr virus (EBV), Encephalomyocarditis virus (EMCV), Foot-and-mouth disease virus (FMDV), IBV Infectious bronchitis virus (IBV), Bovine viral diarrhea virus (BVDV), Classical swine fever virus (CSFV), as well as some plant viruses [16]. As RACK1 is a required protein that aids IRES-based cap-independent mRNA translation from several viruses, functional inhibition of RACK1, without any detrimental effect to the host cells [3], holds promise for the development of a HTA against a broad range of viruses.

Herpes simplex virus (HSV) belongs to the alpha herpesvirus subfamily and is a common human pathogen that causes recurrent infections through its ability to establish a latent state in sensory ganglia after primary epithelial infection [for a general review, see reference [17]. HSV infects a large population (over 500 million worldwide) and is associated with a variety of diseases, including genital herpes, most cases are caused by HSV-2 and partly by HSV-1 [18], ocular herpes, induced by HSV-1 and a leading cause of blindness worldwide [18], and neonatal herpes, which is usually an infectious consequence of either HSV-1 or HSV-2 via vertical transmission [18–20]. One of the characteristics of HSV infection is its ability to latently infect neurons so that it can be reactivated, thereby causing recurrent infections [21]. Although the clinical symptoms of HSV-caused diseases can be controlled with antiviral drugs (acyclovir and valacyclovir), these drugs are not strong enough to stop subclinical transmission [20, 22]. In addition, resistances to acyclovir and valacyclovir frequently occur. No prophylactic or therapeutic vaccine against HSV is available. Therefore, more effective preventive and/or therapeutic drugs against the HSV infection are needed.

Here we report the discovery of functional inhibitor compounds for RACK1 and the effectiveness of these compounds against Herpes Simplex Virus-1 (HSV-1) proliferation. This strategy of targeting a host factor, instead of the virus directly, potentially circumvents the damaging effect of resistance developed by viruses after the prolonged use of promising antiviral drugs.

## RESULTS

Post-translational modifications such as tyrosine phosphorylation and protein sumoylation have been implicated in the regulation of RACK1 function in various organisms [23, 24]. Mutagenesis work has identified Tyr246 as a potential phosphorylation site and has suggested a correlation between enhanced tyrosine phosphorylation of RACK1 and binding of RACK1 to Src tyrosine kinase [23]. In plants, tyrosine phosphorylation by dual-specificity serine/threonine/tyrosine kinase has been proposed [25]. The Y248 residue of *Arabidopsis* RACK1A protein is the conserved residue that corresponds to the human RACK1 Y246 site in a sequence alignment [26]. The RACK1A crystal structure showed that the side chain of Tyr248 (Y248) in the RACK1A protein is located at the end of the loop connecting β-strands A and B of blade 6, and is fully exposed to the solvent making it easily accessible for modification [26]. Recently, it was shown that mutagenesis of Y248F abolished the homo-dimerization potential of RACK1A proteins [27]. Moreover, while wild-type RACK1A scaffold protein, when used as bait, could interact with almost 100 different proteins, RACK1A-Y248F bait failed to interact with any protein [27], implicating the residue in the functional regulation of RACK1 protein. It is quite possible that post-translational modifications, like Y248 phosphorylation, are needed to stabilize the RACK1A protein [28–32]. Considering that RACK1 proteins homo/hetero-dimerize, it is hypothesized that the dimerization status of RACK1 proteins, dependent on Y248 residue phosphorylation, may dictate the regulation of specific signaling pathways by fine tuning affinities for interacting proteins [28].

As viruses require host factors to translate their transcripts, targeting the host factor(s) offers a unique opportunity to develop novel antiviral drugs. In addition, the low variability of host factors targeted by host-targeted antivirals (HTAs) results in a high genetic barrier to resistance [33]. In this regard, we report here the identification of inhibitor compounds for the host protein RACK1, a protein that is utilized by many viruses for their own proliferation. The requirement for the Y248 residue phosphorylation for both homo-dimerization and interaction with diverse proteins has led us to target the site for isolating small compounds that could bind the Y248 pocket and thus prevent its phosphorylation. We hypothesized that functional inhibitor compounds of RACK1 may prevent the proliferation of those viruses that use host RACK1 protein for their mRNA translation.

### SD-29 is identified as a potent binder to the RACK1A Y248 phosphorylation pocket

**Figure 1:**
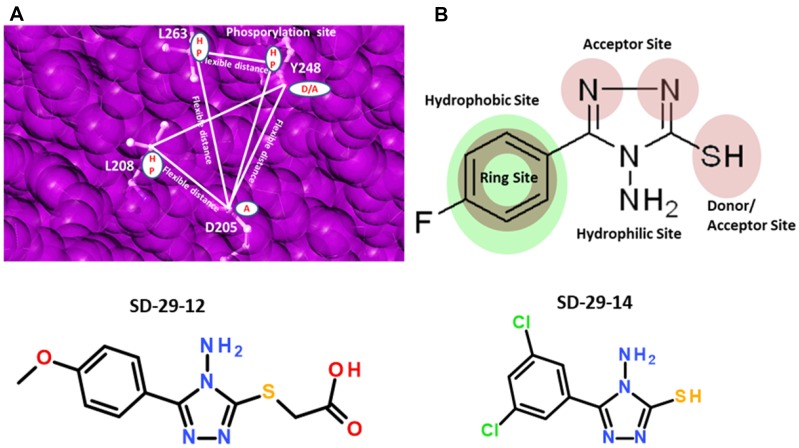
(**A**) Shown are sample two receptor-based three-point pharmacophore models generated on the RACK1A phosphorylation site with exclusion spheres colored pink, geometric and distance constraints (flexible) shown as lines and filled white circles as centers. HP-hydrophobic; D-donor; A-acceptor. (**B**) Ligand-based pharmacophore model generated on SD-29 with pharmacophore constraints acceptor, donor, hydrophobic ring, and hydrophilic sites represented filled circles. Structures of compounds SD-29-12 and SD-29-14 are shown.

**Figure 2:**
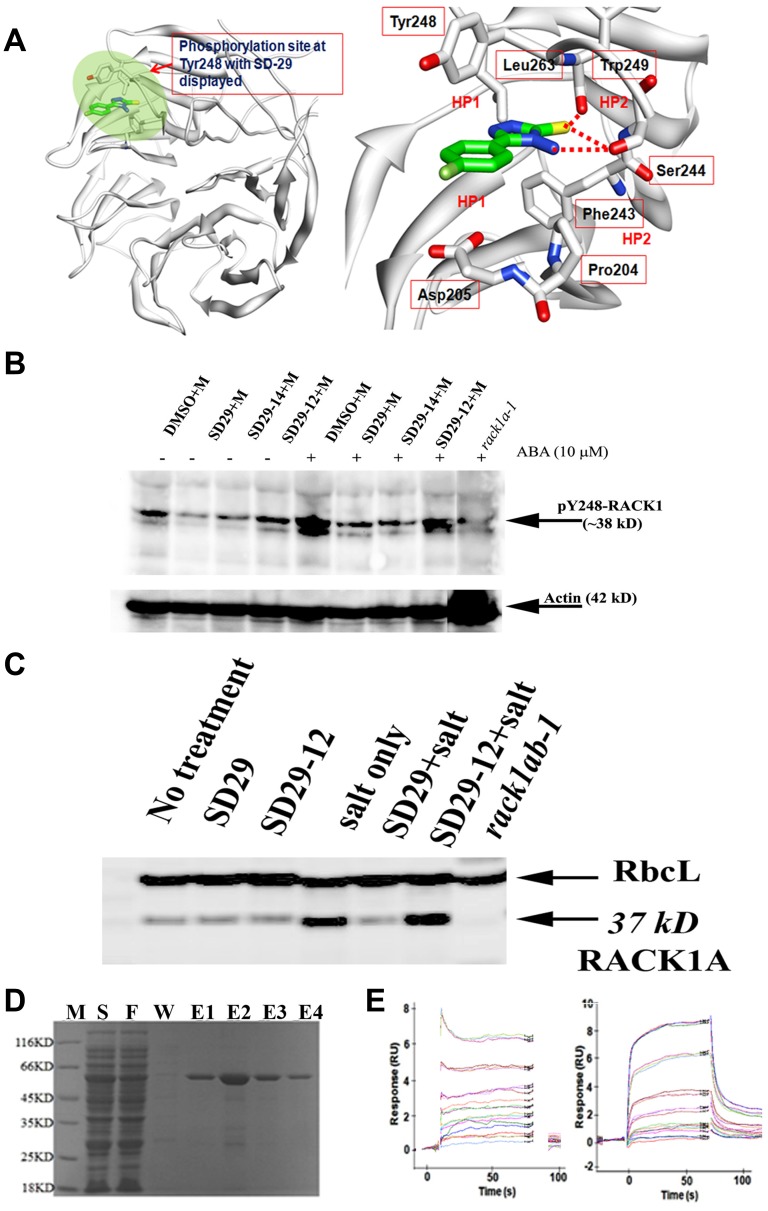
(**A**) Docked Model of RACK1A with SD-29 at the Y248 phosphorylation site. (left panel) Modeled structure of RACK1A with SD-29 (carbon in green) docked into it. The targeted binding pocket is highlighted in green. RACK1A is shown as ribbon model (white). (right panel) Detailed view of the SD-29 (carbon in green) interaction with RACK1A site pocket. The residues interacting with SD-29 are shown in a ball-and-stick model. Hydrogen bonds are shown as red broken lines. SD-29 binding site is surrounded by both hydrophobic (HP1) and hydrophilic residues (HP2). The structural model of ‘SD-29’ with RACK1A showing hydrogen bonds with Ser244, Trp249 and hydrophobic interactions with Tyr248, Phe243, Pro204, Leu 263and Trp249 residues. (**B**) RACK1 functional inhibitor compounds inhibit stress hormone induced RACK1A Y248 phosphorylation. One-week old *Arabidopsis* seedlings were treated with 10 μM of stress hormone Abscisic acid (ABA) in the presence/absence of the inhibitor compounds for 12 hours in a growth chamber (overnight) at 22^°^C. Lysates were probed with an antibody raised to detect phosphorylated Y248 residue of RACK1A protein in *Arabidopsis*. Lysates from a *rack1a-1* knock-out mutant seedlings grown and treated similarly as the Wild Type seedlings were used as negative control. The compounds were dissolved in DMSO (D) and ABA was dissolved in methanol (M). The lower panel shows the same membrane stripped with stripping buffer and then probed with an *Arabidopsis* Actin antibody to show the loading control. (**C**) Salt stress-induced upregulation of RACK1 expression was inhibited by SD-29. The abundant leaf protein Rubisco large subunit (RbcL) was used as loading control for the blot. The 37kD RACK1 band was absent from the genetic knockout of RACK1 plants (double mutant-*rack1ab* lane). (**D**) Purified RACK1 protein on a SDS-PAGE gel. *E. coli* BL21(DE3) host strain was transformed with recombinant plasmid containing rice RACK1 (Chr05 Os05g47890) cDNA with a 3’ His tag. PMSF-induced bacterial lysate eluted from the glutathione-resin column was resolved by the SDS-PAGE electrophoresis for purity check. Lane M: Protein Marker; Lane S: Supernatant; Lane F: Flow through of supernatant; Lane W: Wash; and Lane E1~4: Elutions. (**E**) In the SPR assay, SD-29 (left panel) and SD-29-12 (right panel) bind directly to immobilized RACK1A on the surface of the chip via similar patterns, as evident in the sensogram. SD-29 (left panel) and SD-29-12 (right panel) were separately injected three times on the CM5 chip at 0, 1.56 μM, at 3.13 μM, 6.25 μM, 12.5 μM, 25 μM, 50 μM, and 100 μM (top sensor) concentrations (left panel) and at 3.13 μM, 6.25 μM, 12.5 μM, 25 μM, 50 μM, and 100 μM (top sensor) concentrations (right panel).

By the implementation of a structure based drug design approach, we identified the best-fitting candidate RACK1A Y248 pocket binding small compound- SD-29 the 4-amino-5-phenyl-1,2,4-triazole-3-thiol class of compounds and its analogs are used to provide precise regulation of reported RACK1 mediated specific viral proliferation. To isolate the best-fit compounds, we used the multi-step screening approach, in which each step acts as a filter comprised of protein conformation sampling to account for flexibility of unbound proteins prior to docking simulations. To generate the pharmacophore model, the relative positions of the donor/acceptor sites and hydrophobic centers were used as potential pharmacophore sites. The acceptor (A), donor (D), hydrophobic sites, and negative/positive centers were defined with various macro, spatial and constraints features with exclusion spheres centered on the receptor site. A pharmacophore match search was performed on a small molecule database that contains five million commercially available compounds, including natural product compounds. Figure 1A shows a receptor-based pharmacophore model generated on the Y248 RACK1A site (phosphorylation site) with exclusion spheres. To get appropriate docking, the exclusion spheres were used up to 8Å region from the binding site region. Using this strategy, we identified a candidate compound, SD-29 that putatively binds to RACK1A Y248 (Figure 2A). Using the identified SD-29 structure, a ligand pharmacophore model with various macros, spatial and constraints features defining centroid, acceptor (A), donor (D), and hydrophobic sites/centers was developed to aid in further identification of additional compounds (Figure 1B).

Figure 2A shows the docked model of RACK1A with the most potent small compound (SD-29) at the Y248 phosphorylation pocket. The detailed view of SD-29 (carbon in green) interaction with the RACK1A binding site, indicates that SD-29 potentially form hydrogen bond with Ser244 and Trp249, and maintains hydrophobic interactions with Try248, Phe204, Leu263, and Trp249 residues. In order to obtain *in vitro* confirmation of the binding of the isolated compounds to the purified RACK1A protein, recombinant RACK1A protein from rice was raised in *E. coli* cells (Figure 2D). The small compound SD-29 and its structural analog (SD29-12) were used to evaluate their binding potential with the recombinant RACK1A proteins. The compounds were examined for their ability to interact directly with wild-type recombinant protein immobilized on the Surface Plasmon Resonance (SPR) chip. The SPR assays showed that the compounds SD-29 and SD29-12 bound strongly to recombinant RACK1A protein with an KD_50_ value of 42 μM and 58 μM, respectively (Figure 2E). This *in-vitro* confirmation of binding of the compounds to RACK1A protein has led to us to investigate whether the binding has any effect on the reported host factor RACK1 mediated virus proliferation.

### Compounds can potentially inhibit stress-induced RACK1A Y248 phosphorylation

In order to evaluate whether SD-29 can specifically inhibit RACK1A tyrosine phosphorylation, particularly on the Y248 residue, an anti-phospho-Y248-RACK1 antibody was raised commercially (Creative Diagnostics, NY). To ascertain whether the antibody only recognizes the *Arabidopsis* Y248 in the RACK1A protein, and not in the RACK1B or RACK1C proteins, the RACK1A-specific phospho-peptide (10 residues) was adsorbed against the non-phosphorylated RACK1A, RACK1B and RACK1C peptides and the adsorbed phospho-peptide was used as the antigen in rabbit. RACK1A Y248 phosphorylation has previously been identified as a key event needed for RACK1 mediated scaffolding activities by regulating protein-protein interactions in plants [27, 28]. The corresponding residue in human RACK1 is also a key requirement for scaffolding activities needed at the receptor level [23]. As RACK1A is known to regulate the diverse stress responses in plants [34], we investigated the role stress hormone abscisic acid on the RACK1A Y248 phosphorylation. It is known that ABA mediated signaling pathways regulate diverse biotic and abiotic stresses including salt and drought stresses [35] and RACK1A has been implicated in the ABA mediated stress pathways in *Arabidopsis* [36]. Therefore, we used young *Arabidopsis* seedlings treated with/without ABA for 12 hours to mimic stress conditions. As a control, we used lysates from the *rack1a-1* knock-out seedlings. While the actin antibody shows the almost equal loading, the presence or absence of ABA showed a significant role on the RACK1A Y248 phosphorylation (Figure 2B). The lane with ABA but no inhibitor compounds clearly showed that the Y248 residue of RACK1A proteins were highly phosphorylated, while the inhibitor compounds (SD 29 and SD 29-14) prevented the ABA-induced Y248 phosphorylation which showed almost as the same level without the stress hormone present (Figure 2B). Note that, the antibody was raised by using RACK1A Y248 phosphorylated peptide as immunogen and by adsorbing against the non-phosphorylated RACK1A, RACK1B, and RACK1C peptides. Comparing with the negative control and considering the scheme to raise the antibody, it can be asserted that the SD29, SD29-14, and SD29-12 to a lesser extent, can potentially inhibit the stress induced Y248 phosphorylation.

The reported positive expression of RACK1A protein under salt stress condition in *Arabidopsis* has led us to examine the RACK1A expression in the presence and absence of the inhibitor compounds during salt stresses. We determined that the RACK1A protein expression was specifically modulated by the inhibitor compounds when the lysates with salt or without salt in the presence and absence of the small compounds were probed with an antibody raised by using the full length RACK1A protein as antigen (Agrisera, Vännäs, Sweden, Figure 2C). When challenged with salt stress, SD-29, but not its analog SD29-12, inhibited RACK1 protein expression. However, whether the effect of SD29-12 is *Arabidopsis* salt stress specific condition needs further experimental evidence. As the antibody cross-reacts with all three RACK1 isoforms in *Arabidopsis*, we used the double knockout (*rack1ab*) as a negative control on the blot (triple mutant is lethal at the early seedling stage). The large subunit of the abundant leaf protein rubisco (RbcL) was used as loading control for this blot. As the results support the *in-silico* based prediction, we set out to investigate the effect of the compounds on the mammalian RACK1 based virus proliferation.

### HSV-1 infection induced RACK1 expression.

**Figure 3:**
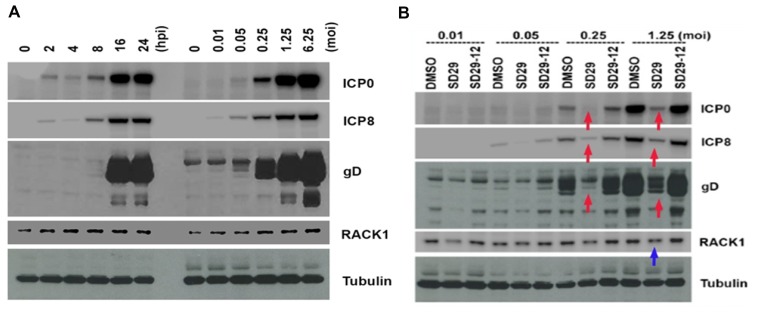
Effects of the compounds on HSV-1 protein expression. (**A**) Time and concentration dependent virus protein expression to deduce optimum drug treatment regimen. Left panel shows the time dependent HSV-1 virus protein expression after infection with HSV-1 17 at MOI 1.25. Right panel shows the same virus protein expression under different concentrations of virus starting from 0.01 to 6.25 MOI. (**B**) Hep-2 cells were pre-treated with the compounds for 24 h and three different virus proteins (ICP0, ICP8, and gD) were assayed 24 h post infection with different titers of HSV-1. Red arrows show the downregulated virus proteins and blue arrow shows the downregulated RACK1 protein. Tubulin expression was used as the loading control for the blots.

Since RACK1 is an important factor for protein translation, we wondered whether RACK1 expression is regulated by viral infection. We tested this hypothesis in a HSV-1 infection system in the HEp-2 cell line. This cell line was selected because the human RACK1 target site Y246 shows strong similarity to the *Arabidopsis* Y248 pocket. First, we infected HEp-2 cells with HSV-1 at an MOI of 1.25. The whole cell lysates were collected at different time points as indicated in Figure 3A. The samples were run on an SDS PAGE gel and the proteins were transferred to a membrane which was blotted with antibodies for viral proteins (ICP0, ICP8, and gD) and for cellular proteins (RACK1 and tubulin). RACK1 was induced at the very beginning of the viral infection (2 hpi), as can be seen by comparing the levels of RACK1 after viral infection to that of 0 hpi (Figure 3A). The increasing protein level of RACK1 reached its peak at 8 hpi. We then wondered whether RACK1 upregulation is related to the viral concentration. For that purpose, we infected HEp-2 cells with different MOIs of HSV-1 for 24 hours to examine the levels of RACK1. RACK1 levels were increased after viral infection and the increase in RACK1 was proportionally associated with the MOI of virus (Figure 3A). Therefore, HSV-1 infection induces the production of RACK1 at very early time points after infection and the level of RACK1 upregulation is dependent on viral dose. In addition, as expected all the viral proteins were up-regulated with increasing viral loads and at increasing time-points (Figure 3A). The results helped establishing the concentration and time-points needed to see whether the compounds have any effect on the viral proliferation that is marked by the increasing synthesis of viral proteins.

### SD-29 has repressive effects on viral protein production

To determine whether the small compound SD-29 that binds to RACK1A protein and inhibits Y248 phosphorylation, has any effects on viral protein production, we treated HEp-2 cells with SD-29, its analogue (SD29-12), or DMSO for 24 hours at 100 μM, and then the cells were infected with different MOIs of HSV-1 (Figure 3B). Twenty-four hours after infection, the whole cell lysate samples were subjected to western blot assay to examine the viral and cellular proteins. SD-29 reduced the production of RACK1 as compared to the DMSO or SD-29-12 control treatment groups (Figure 3B). Clearly, viral protein levels from SD-29-treated HEp-2 cells were lower than that of DMSO- or SD-29-12- treated HEp-2 cells. We examined three HSV-1 proteins: ICP0 is an immediate early (IE) protein, ICP8 is an early (E) protein, and gD is a late (L) protein. Therefore, our results demonstrated that SD-29 repressed HSV-1 protein production, which can effectively inhibit virus proliferation in the cell line.

### Compounds effect on HSV-1 gene transcription

**Figure 4:**
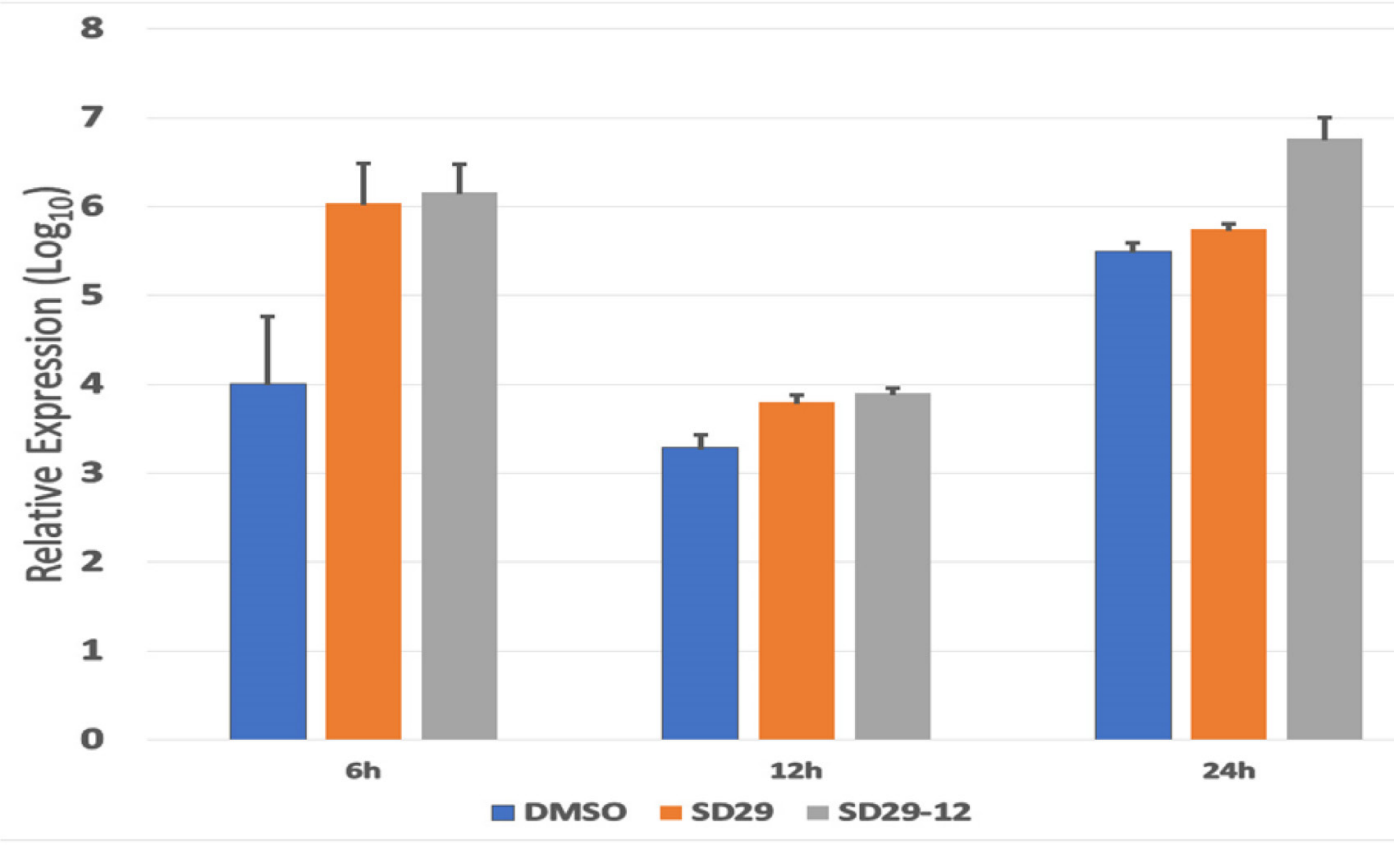
Real-time PCR analysis of HSV-1 *ICP0* **expression in the Hep-2 primary treated with the indicated inhibitor compounds and DMSO (as control) for the indicated time points.** The total transcript levels were quantified using actin expression as an internal control. The cDNA obtained immediately after the virus and drugs transfection was set as a baseline value. The normalized expression values were transformed to the 1og_10_ value. The data plot represents mean ± standard error calculated from three replicates.

Since RACK1 is an important chaperone protein for ribosome function in mRNA translation, we assumed that SD-29 might affect viral gene expression only at the translational level. The compounds were developed to specifically inhibit the RACK1 protein; therefore, it is not expected to affect the mRNA production of assayed proteins (Figure 3). To investigate whether SD-29 is functioning as expected, the mRNA expression level of the ICP0 gene was measured in qPCR assay (Figure 4). Though initially after the infection, the HSV-1 *ICP0* transcript level was upregulated to some extent by the compounds, over time the effect subsided and fall at the same level as with the DMSO treated samples (Figure 4). Note that the assayed gene *ICP0* is an immediate early gene and the ICP0 protein is capable of transactivating promoters from all three kinetic classes of HSV-1 genes, including immediate-early, early and late. Therefore, high level of transcripts at the early stage of infection may reflect an adaptive response to the challenge by the compounds. The compound treated (SD-29, SD29-12) and non-treated (DMSO) samples essentially showed the same level of *ICP0* mRNA at 12 and 24 hpi after showing slight upregulation initially (Figure 4). The results establish the specificity of the SD-29 in inhibiting the level of key proteins needed by the HSV-1 to proliferate while not affecting mRNA levels.

### SD-29 inhibits viral proliferation

To assess the efficacy of SD-29 on the HSV-1 proliferation, a plaque assay was performed. We wanted to quantitatively assess whether SD-29 could inhibit viral replication. Because HSV-1 was able to enter cultured HEp-2 cells, we evaluated whether this entry led to productive virus replication. The cytopathic effect in the form of plaque formation increased significantly over time in virus-infected HEp-2 cells treated with vehicle, as seen in the Table 1. As shown in Table 1, inoculum harvested from infected HEp-2 cells treated with DMSO produced a larger number of plaques 24 hours post infection. In contrast, cells infected with identical doses of the same virus and treated with SD-29 failed to produce significant infectious virions. These results, together with those of the entry assay, show that treatment with SD-29 led to the inhibition of a productive infection.

**Table 1:** Plaque formation unit (pfu) assay

	DMSO	SD-29	SD29-12
0 hpi	0 ± 0	0 ± 0	0 ± 0
6 hpi	3.2 ± 0.08 ×10^2^ PFU/mL	0 ± 0	3.8 ± 0.07 ×10^2^ PFU/mL
12 hpi	2.6 ± 0.03 ×10^3^ PFU/mL	1 ± 0.08 ×10^2^ PFU/mL	3.6 ± 0.04 ×10^3^ PFU/mL
24 hpi	1.9 ± 0.02 ×10^6^ PFU/mL	1.8 ± 0.04 ×10^5^ PFU/mL	2.6 ± 0.03 ×10^6^ PFU/mL

Confluent monolayers of HEp-2 cells were infected with serially diluted HSV-1 virus and were fixed and Giemsa stained at 0, 6, 12, and 24 hr post infection. The numbers of plaques were visualized. The number of plaques formed post infection decreased in the presence of SD-29 in a time-dependent manner.

In order to assess whether the compounds are producing any toxicity that can led to the cell deaths, we evaluated the cell viability by the trypan blue exclusion method. As long-term inhibition of RACK1 expression has been reported to cell cycle arrest, we limited our experimental treatments to 24 h and evaluated the cell viability of almost full confluent cells after 24 h of compound treatment. It can be seen in Table 2, the compounds at 24h post-treatment did not cause any major cell viability problem as with or without the compounds, the cells maintained over 90% viability.

**Table 2:** HEp-2 Cell viability after 24-hour treatment with 10 or 100 μM concentration of the inhibitor compounds

Compounds	Mean	Std error (±)
DMSO (Control)	96.0	4.18
SD-29 (100 μM)	92.7	2.38
SD-29 (10 μM)	94.0	5.79
SD29-14 (100 μM)	93.7	2.17
SD29-14 (10 μM)	97.5	2.06
SD29-12 (100 μM)	97.7	0.43
SD29-12 (10 μM)	92.7	3.96

The viability of cells was measured with Trypan blue exclusion assay in a Cellometer (Nexcelom, Lawrence, MA). The percentage survival of the compound treated cells was evaluated along with the DMSO treated cells. The values represent the mean ± SE of three separate well based replicates.

### Visualization of the compound induced inhibition of HSV-1 proliferation

**Figure 5:**
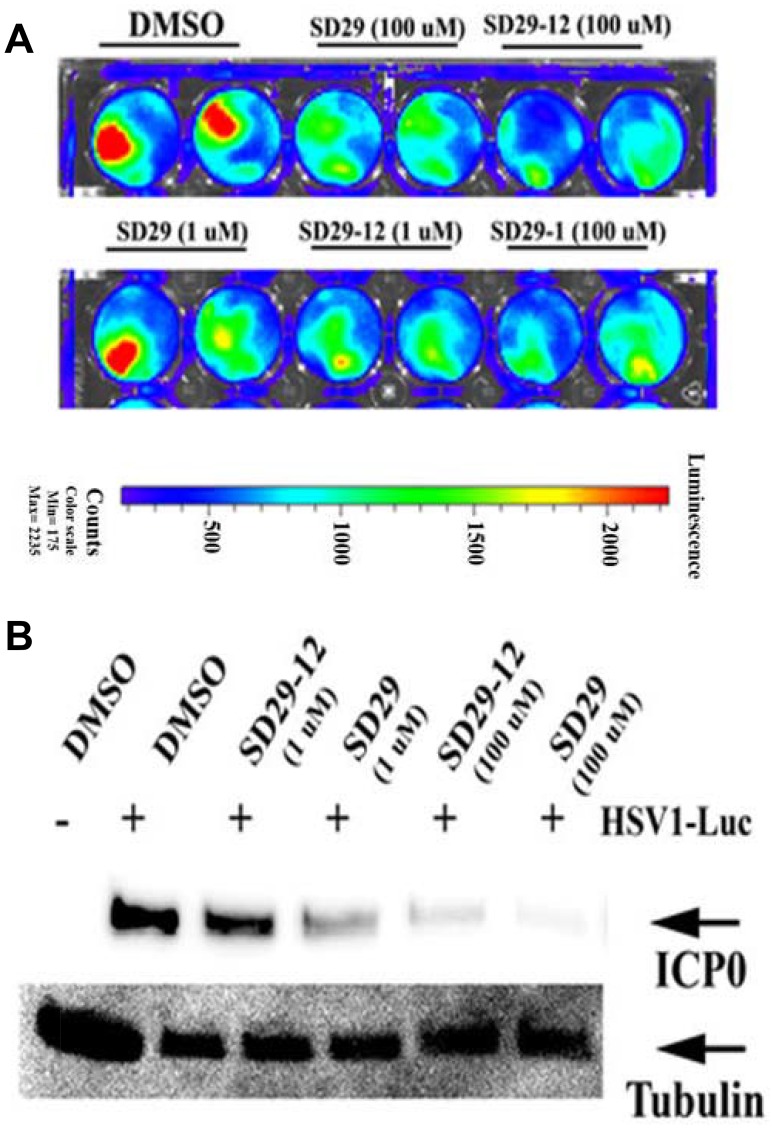
Co-treatment of drugs with virus increases efficacy. (**A**) The effect of drugs of indicated concentration after 24-hour post-infection with HSV1-Luc (4 × 10^6^ pfu/ml media) and the same treatments are shown in duplicates. (**B**) The HSV-1 major structural protein ICP0 expression was assayed from the 48-hour post infection samples as shown in panel (**A**). The expression of tubulin was used as loading control (lower panel).

To visualize the effect of the drugs on the proliferation of HSV-1, a luciferase tagged HSV-1 F strain expressing luciferase (R8411 mutant) under the control of ICP27 promoter was obtained [37]. The co-treatment of the drugs and the virus at the same resulted in a dose dependent lowering of luciferase signals- indicating an inhibitory effect of the compounds (Figure 5A). The same samples after incubation of 48 h were used to assay for HSV-1 ICP0 and RACK1 protein expression (Figure 5B). Depletion of RACK1 by the compounds correlated with the depletion of the viral ICP0 protein confirming earlier results shown in Figure 3B.

### An analog of SD-29 reveals better efficacy in the inhibition of HSV-1 proliferation

**Figure 6:**
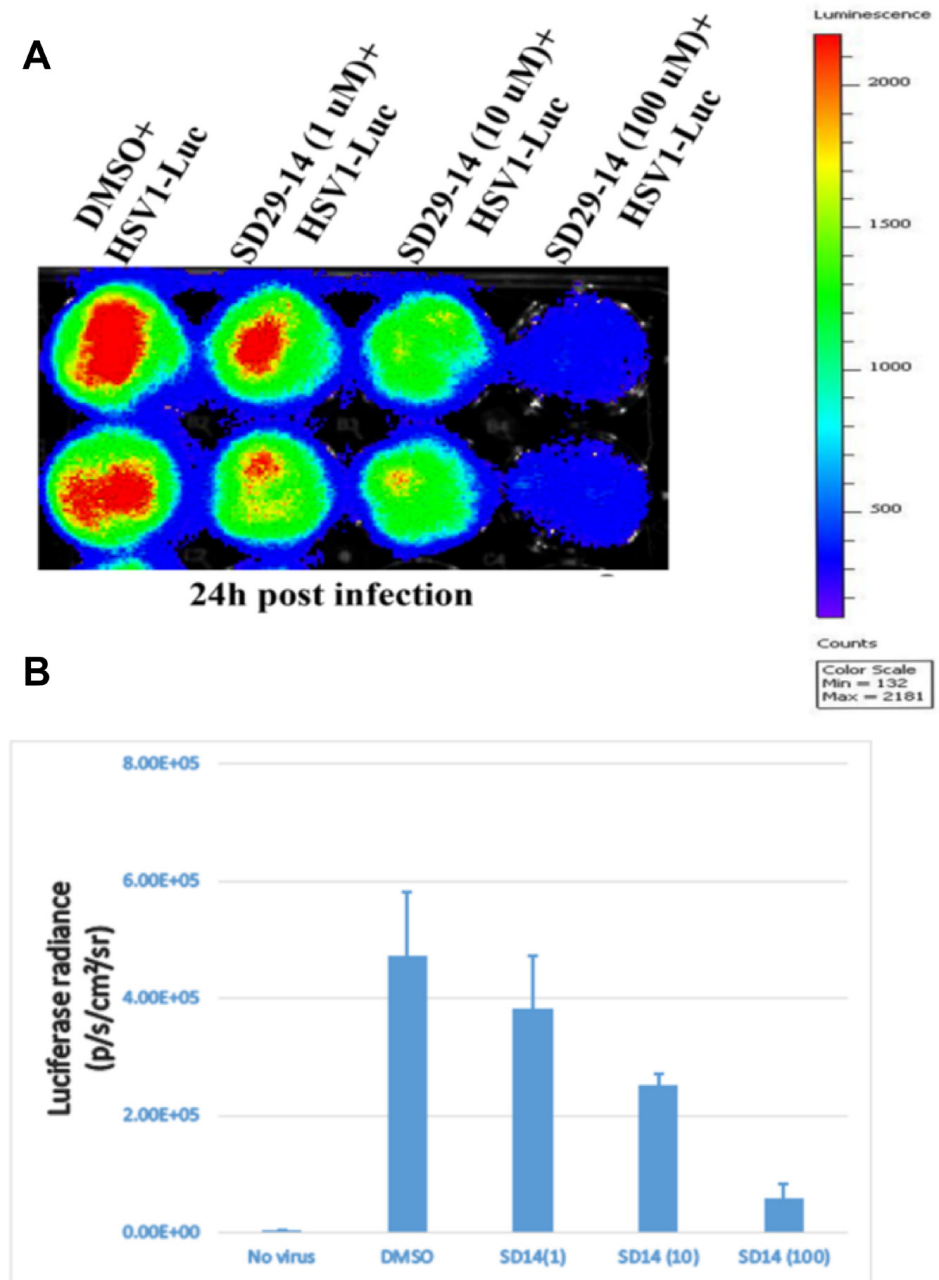
Visualization of the RACK1 inhibitor induced inhibition of HSV-1 proliferation in the HEp-2 cells. The HSV-1-Luc virus and the indicated concentration of compounds were added and incubated for 24 h (panels **A** and **B**) samples. The luciferase signals were imaged and quantified in a Perkin Elmer IVIS Spectrum Imaging system. (**A**) The inhibitor compounds effectively inhibited the HSV-1 proliferation as can be seen in a dose dependent reduction in the luciferase signal (red). (**B**) Quantification of the luciferase signal from the samples in panel (**A**). Three replicates from two separate experiments were combined to generate the average and the standard error bar.

Through similar docking experiments, an analog with chloro at the meta positions of the phenyl ring (SD29-14) instead of the mono-substituted (fluoro) at the para-position of the phenyl ring (SD-29) was isolated. SD29-14 analog showed strong inhibition of HSV-1 proliferation in a dose dependent manner (Figure 6A). While SD-29 showed much less efficacy at the 1μM concentration, SD29-14 significantly inhibited the HSV-1 proliferation as evident by the lower luciferase signals. The luciferase signals were measured quantitively and showed dose dependent inhibitory effect of the SD29-14 on the HSV-1 proliferation (Figure 6B). Availability of the compounds with better efficacy will allow application of the compounds at lower concentration which will circumvent any toxicity that higher concentration of compounds may pose. In addition, the better efficacy will allow the compounds to be tested against other IRES utilizing human pathogenic viruses as well.

### SD29-14 inhibits ICP0 expression

**Figure 7:**
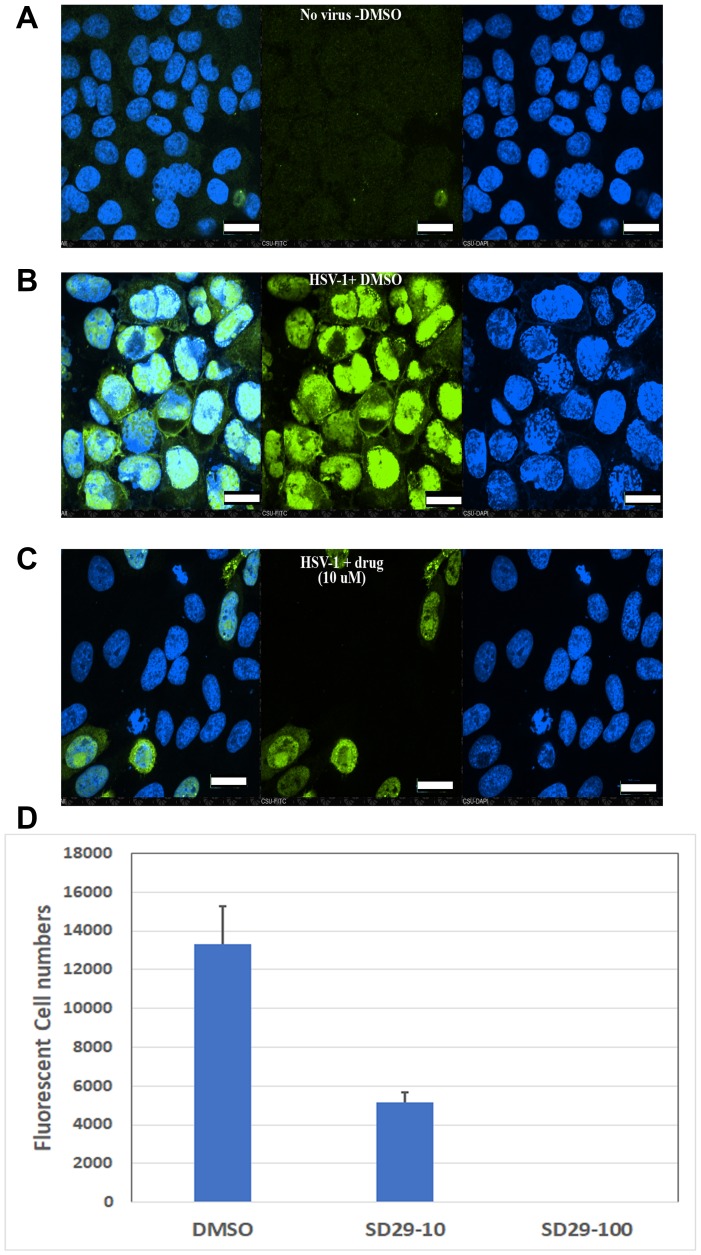
Immunofluorescent evidence for HSV-1 proliferation inhibition by the RACK1 inhibitor compound SD29-14. The HEp-2 cells were infected with HSV-1 at an MOI of 1.0 for 30h along with the indicated compounds and control. The no-virus control (**A**), vehicle DMSO treated (**B**) and SD29-14 10 μM treated (**C**) cells were fixed with 4% paraformaldehyde and stained with anti-ICP0-FITC (green) and DAPI for DNA (blue) in the nucleus. The slides were observed under a Nikon confocal microscope (60 × magnification lens) and pictures were taken to show infected cells (green) and total cells (DAPI). All scale bars correspond to 20 μm. The imaging experiments were performed three independent times, and the results shown represent one of the three experiments. (**D**) Quantification of fluorescent cells by Image based cell sorting in a Cellometer (Nexcelom Vision). The Y-axis shows the total fluorescent cells per ml. The cells were prepared by immunostaining with ICP0 antibody after 24-h incubation with the indicated compounds. The total cell counts were 1.01 × 10^6^/ml, 4.69 × 10^6^/ml, and 5.36 × 10^6^/ml for DMSO, SD29-14 (10 μM), and SD29-14 (100 μM) respectively. The numbers show the mean ± SE of three replicates.

Our results provide evidence for the efficacy of the SD 29-14 against the HSV-1 proliferation, however, to further confirm this we set out to demonstrate via direct visualization the effect of the drugs on the proliferation of HSV-1 in living cells. In this regard, immunofluorescence studies were undertaken where HSV-1 ICP0 protein expression levels were visualized with or without the compounds with higher efficacy (SD29-14) in the Hep-2 cell line. We found that without treatment of the compound, the HSV-1 transfected cells express the viral ICP0 protein uniformly in the nucleus as it overlaps with the nuclear stain DAPI (Figure 7B), while treatment with 10 μM concentration of the compound effectively eliminated any expression of ICP0 protein from the cell (Figure 7C). The immuno-stained cells without any virus infection were used as a negative control (Figure 7A). Note that the absence of FITC stain is not due to the toxicity induced cell death. The DAPI staining of the same cells indicates that viable cells were present, but due to the presence of the compound a significant inhibition of viral proliferation was observed. The results as visualized by the FITC tagged secondary antibody indicate that HSV-1 proliferation is inhibited by the higher efficacy compound SD29-14 with a lower effective concentration.

### Comparison of SD29-14 efficacy with known anti-herpes drug acyclovir

**Figure 8:**
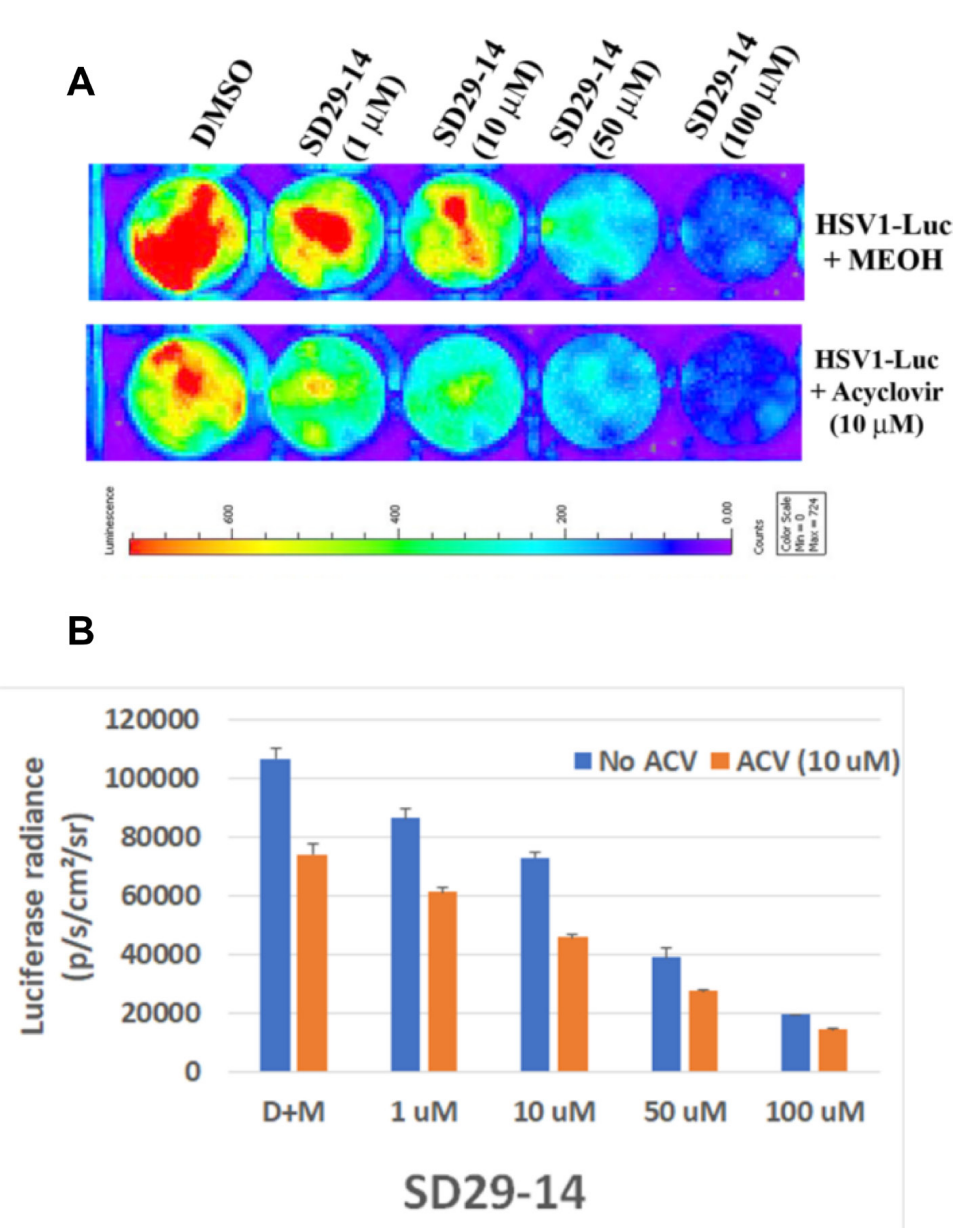
Efficacy of SD29-14 compared with anti-herpes drug acyclovir. (**A**) Hep-2 cells were incubated with the indicated compounds in the presence of HSV1-Luc virus at a concentration of 4 × 10^6^ pfu/ml media. (**B**) Quantification of the luciferase signal from the samples in panel (**A**). Signals from three replicates from two separate experiments were combined to generate the average and the standard error bar.

Acyclovir is the major anti-herpes drug on the market and evaluated the SD29-14 efficacy with that of acyclovir. As can be seen from Figure 8, application of SD29-14 could effectively inhibit the HSV1-Luc proliferation starting from 1 μM concentration (Figure 8A). As very low concentration of acyclovir could not show the significant inhibition of HSV1-Luc (data not shown), we used the concentration of 10 μM at which point the drug showed inhibition of the HSV1-Luc proliferation. Our developed drug appears to show efficacy at slightly higher level than that of acyclovir induced effect. Acyclovir is known to be an inhibitor of viral DNA replication while SD29-14 is not known to regulate the viral DNA replication; therefore, we expected that there will be no interaction between the inhibitory pathways of these two drugs. In the absence of interaction, it is expected that the combinatorial treatment may potentially show synergistic effect in inhibition. Therefore, we treated the virus infected cells with different combination of the drugs. As can be seen from the Figure 8A, a supralinear effect is apparent at all different concentrations of SD29-14 combined with 10 μM of acyclovir. When the luciferase signal is quantified, it shows a dose-dependent inhibition of HSV1-Luc proliferation in all concentrations of the drugs used either in combination or SD29-14 alone (Figure 8B). We believe this combinatorial application will allow a better approach to combat the HSV1 which is increasingly becoming resistant to the drugs available on the market.

## DISCUSSION

Viruses pose a constant threat for all living organisms and they constantly evolve to evade any anti-viral drugs that directly aim to their intrinsic targets. The clinically effective drugs acyclovir and valacyclovir [38] are available to treat HSV-caused diseases, but resistant to these drugs are reported [39]. HSV infection remains an important public health problem causing significant morbidity and mortality [40]. In addition, HSV infections disproportionately affect health disparity populations. Specifically, HSV infections are related to racial-/ethnic-, age-, and gender-associated health disparities [41–46]. More importantly, HSV-1 and HSV-2 infection rates go up more rapidly in minority groups than in Caucasians [46]. Therefore, targeting HSV-caused diseases will significantly contribute to the reduction of health disparities and new drugs developed by this novel strategy will provide essential therapy.

By using a combination of bioinformatics, biochemical, and molecular approaches, here we have developed a class of broad anti-viral compounds which instead of directly targeting the virus, target a host factor utilized by diverse classes of pathogenic viruses. This approach offers a unique avenue for drug development to prevent the pathogenic viruses that hijack molecular machineries from host cells to complete their replication cycle. As a proof of concept, here we presented evidence for the drugs action against HSV-1. RACK1-mediated viral mRNA translation appears to be a crucial step in virus propagation. Thus, RACK1 is established as an effective host target for developing broad antiviral therapy. Interestingly, RACK1 was reported to be the host factor for the HCV IRES-mediated translation where it was shown that RACK1 is not required for the classical cap-dependent translation and inhibition of RACK1 expression in cell culture models by RNAi method did not reveal major toxicity [3]. Similarly, down-regulation of the RACK1 in a human cell line by the compounds identified in this work did not reveal any major toxicity in terms of cell death. Thus, it appears that the administration of the compounds for anti-viral effect will not result in any discernable major side effects that often accompany with diverse drugs in the human clinical trials.

IRES regions have been found in a broad range of viruses including HIV-1, HCV, HSV-1, and Enteroviruses including EV-D68 which has been casually correlated with the recent outbreak of polio like symptoms in children. Availability of a functional inhibitor of the widely conserved RACK1 protein will now allow us to investigate how these compounds can be broadly applied against a wide range of IRES utilizing viruses. Collectively, our data unravel a specific function for ribosomal protein RACK1 in selective mRNA translation of a human virus and uncover a previously undiscovered target for the development of broad antiviral intervention. The identification offers a unique opportunity not only to develop a durable drug but also it can potentially circumvent the resistance often developed for many drugs targeting directly a viral target. Indeed, it is reported that HTAs effectively inhibit HCV escape variants [47, 48], as well as Direct Acting Anti-viral (DAA)-resistant viruses [49]. Furthermore, their complementary mechanism of action results in synergy with DAAs [50]. Given that HTAs interfere with host targets, one potential caveat is greater risk of cellular toxicity as compared to DAAs. Interestingly, our data obtained in cell culture models did not reveal any major toxicity linked to RACK1 inhibition confirming the other genetic studies in many cell cultures. These results have established the RACK1A inhibitor compounds as a new effective anti-HSV-1 drug, which is urgently needed. In addition, we present evidence that combinatorial application of the drug with the existing anti-herpes drug acyclovir may provide a better approach to combat the virus through synergistic action of the drugs. Application of these inhibitor compounds will not only be useful as HTAs, but many other diseases where RACK1 expression has been implicated in disease progression. For example, in cancer metastasis [50–52], acute pancreatitis [53], Cardiovascular disease [54], plant pathogenic viruses using IRES [16], and Leishmaniasis [55], RACK1 expression has been shown to produce poor prognosis. Therefore, chemical inhibitor molecule of RACK1 protein should be effective in alleviating the disease progression in these circumstances as well.

Additionally, *RACK1A* has been shown as the negative regulator of stress hormone abscisic acid (ABA) in the model plant *Arabidopsis* to regulate drought stress responses [26, 31, 34], hence, the availability of the RACK1A inhibitor compound will effectively allow to use the compounds as anti-drought compounds in many crops that maintain essentially the similar *RACK1A* gene sequences.

This work will usher a new avenue to develop effective and durable drugs that can potentially circumvent the selection pressure induced drug-resistance scenarios. Future endeavors to generate next generation drugs targeting the host RACK1 protein with enhanced efficacy will advance this approach to a significantly new level where direct targeting anti-viral drugs are slowly being rendered as non-effective in term of their effective durability

## MATERIALS AND METHODS

### Tissue culture and viruses

HEp-2 cells (ATCC) were used for the infection of HSV-1. The cells were maintained in Dulbecco’s Modified Eagle’s medium (DMEM) supplemented with 10% fetal calf serum (FCS) and 1% penicillin-streptomycin (PS). Wild-type HSV-1 17 was obtained from R.D. Everett and used previously [56].

### Antibodies

The antibodies used for Western blot (WB) and immunofluorescence are listed below. A monoclonal antibody against tubulin (T-9026) was purchased from Sigma–Aldrich (St. Louis, MO, USA; 1:1000 for WB); polyclonal antibody against ICP8, and monoclonal antibodies against HSV ICP0, ICP4, RACK1 and gD were purchased from Santa Cruz Biotechnology, Inc. (Santa Cruz, CA, USA).

### Purification of viruses

Herpes viruses were amplified in Vero cells. The viral supernatant was centrifuged at 8000 g for 20 min to remove cell debris. The clarified medium was transferred into SW27/28 ultra-clear centrifuge tubes that were underlain with 7 mL of 20% sorbitol buffer (20% D-sorbitol, 50 mM Tris-HCl, pH 7.2, and 1 mM MgCl) and centrifuged at 55,000 g for 1 hour. The purified viral pellet was resuspended in PBS. The HSV-1 F strain expressing luciferase (R8411) under the control of the ICP27 promoter was a gift from Bernard Roizman (University of Chicago) and was constructed using an HSV-1 bacterial artificial chromosome (BAC) system described in Horsburgh *et al*. [37].

### Bioluminescence luciferase assay

To 80–90% confluent HEp-2 cells, HSV1-Luc (R8411) was added at a concentration of 2 × 10e^6^ pfu/ml along with indicated concentration of inhibitor compounds. After the described incubation period, luciferase activity was evaluated from bioluminescence images acquired by a Perkin Elmer IVIS Spectrum Imaging system immediately after adding luciferase substrate (D-luciferin, Gold Bio Technology) at a concentration of 400 μg/mL in medium. Bioluminescence intensity in different wells was quantified in units of photons per second per centimeter squared per steradian (p/s/cm^2^/sr) by drawing a polygonal region of interest over the signals in images using Living Image 3.0 software (Caliper Life Sciences).

### Immunoblot analysis

Proteins were separated by SDS-PAGE (25 μg loaded in each lane), transferred to nitrocellulose membranes (Amersham Inc., Piscataway, NJ, USA), and blocked with 5% nonfat milk for 60 min at room temperature. Membranes were incubated overnight at 4°C with primary antibody, followed by incubation with a horseradish peroxidase-coupled secondary antibody and detection with enhanced chemiluminescence (Pierce, Rockford, IL, USA), according to standard methods. Membranes were stripped with stripping buffer (100 mM 2-mercaptoethanol, 2% SDS, 62.5 mM Tris-HCl, pH 6.8), washed with PBS-0.1% Tween-20, and used to detect additional proteins. For *Arabidopsis* lysates one-week old seedlings were treated with 10 uM of ABA in the presence/absence of the inhibitor compounds for 12 hours in a growth chamber (overnight) at 22C mostly in the night dark. Lysates were isolated in lysis buffer (Cell Signaling, Danvers, MA) supplemented with plant protease inhibitor (Sigma-Aldrich), phosphatase inhibitor cocktail A and B (Santa Cruz Biotechnology, Dallas, TX), and N-Ethylamaleimide (Sigma-Aldrich, St. Louis, MO). Twenty five microgram of proteins were loaded on the BioRad’s 4-12% precast Bis-Tris polyacrylamide gel, transferred to a nitrocellulose membrane and then blocked with 5% Bovine Serum Albumin (BSA) for one hour, washed and incubated with the an antibody (1:100 dilution) to detect phosphorylated Y248 residue of RACK1A protein which was raised using the epitope: FSPNR{pTYR}WLCAATEH (Genscript, Piscataway, NJ, USA). To make it specific to RACK1A pY248, it was raised by adsorbing against the RACK1A non-phosphorylated antigen and to a peptide antigen with sequence FSPNRYWLCAATEN specific for the *Arabidopsis* RACK1B and RACK1C proteins. A rabbit secondary antibody (1:5000) was used. BIORAD’s Clarity ECL substrate was used to visualize the bands. For loading control, the same membrane stripped in mild stripping buffer and then probed with an *Arabidopsis* Actin antibody (Sigma-Aldrich, St. Louis, MO) to show the loading control.

### RNA isolation and real-time RT-PCR

Following instructions of the manufacturers, total RNA was isolated using Tri Reagent^®^ (Ambion, Inc., Austin, TX). To quantitatively examine the mRNA level of HSV-1 ICP0 from the infected cells, real-time RT-PCR was undertaken using the QuantiTect SYBR Green RT-PCR kit (QIAGEN, Valencia, CA). The primers for HSV-1 ICP0 were: forward-5′-CTGCGCTGCGACACCTT-3′ and reverse-5′-CAATTGCATCCAGGTTTTCATG-3′; and the primers for beta-actin (as control) were: forward-5′-GGTTCCGATGCCCTGAGGCTC-3′ and reverse-5′-ACTTGCGGTGCACGATGGAGG-3′. 1 μg of total RNA and 0.2 μM of sense and antisense primers (amplifying the RNA fragment in NS5 location) were used in a final 25 μl master mix volume. PCR reactions consisted of 50 cycles with the following optimal conditions: 94° C for 20 s; 50° C for 1 min; 72° C for 30 s; and an optimized collection data step, 80° C for 5 s. Fluorescence (captured at 80° C) was determined to be absent from the signal generated by primer dimers. Data were collected and recorded by the BIORAD CFX Manager software. The relative quantity of the ICP0 transcripts at the indicated time points was normalized to the relative quantity of the reference gene actin at the same time points and then the log_10_ copy number of the normalized RNA transcripts were calculated and presented on a bar chart. A melting temperature curve analysis was obtained by measuring (after the amplification cycles) the fluorescence during a period of warming from 60° C to 95° C.

### Plaque formation unit (pfu) assay

The viral plaque assay was performed as previously described [56] with slight modifications. The HSV-1 strain 17 was diluted serially in a volume of 1 mL. Then 300 ul of the virus was added onto confluent HEp-2 cell monolayers in 12-well plates; each dilution had 3 wells. After adsorption for 2 h, the medium was removed and the cells were washed twice with serum-free DMEM and overlaid with phenol-free DMEM containing 5% FCS, 0.5% low-melting point agarose (GIBCO), and 1% penicillin-streptomycin. The cells were incubated at 37° C for 48 hours. Neutral red was added on the agarose to stain the live cells. The plaques were counted and reported as pfu per mL. Mean pfu was determined after averaging the number of pfu from different dilutions. Student’s *t*-tests were used to statistically analyze differences between the groups; a *p*-value lower than 0.005 was used as the threshold for a significant difference.

### Immunofluorescence and confocal microscopy

Hep-2 cells were grown to about 80% confluency in 12 well plate and the cells were co-infected with HSV-1 at an MOI of 1.0 for 30 h along with the indicated inhibitor compounds and control. The cells were fixed at room temperature with 4% paraformaldehyde-PBS for 15 minutes and then permeabilized at room temperature by 0.5% Triton X-100–PBS (pH 7.4) for 10 min. The cells were then washed 3X with Ice cold PBS. The cells were Incubated with 1% BSA, 22.52 mg/mL glycine in PBST (PBS+ 0.1% Tween 20) for 30 min to block unspecific binding of the antibodies. The cells were Incubated cells in the 1:500 diluted anti-ICP0 antibody in 1% BSA in PBST in a humidified chamber overnight at 4° C. The cells were washed three times in PBS, 5 min each wash and then Incubated cells with the anti-mouse FITC conjugated secondary antibody in 1% BSA for 1 h at room temperature in the dark. After washing three times with PBST for 5 minutes each, the cells were mounted on slide using Prolong Slowfade Gold with DAPI (4=,6=-diamidino-2-phenylindole; Invitrogen), and confocal analysis was performed using Nikon CSU series Spinning Disk confocal microscope. Images were taken to show infected cells (green) and total viable cells (with DAPI stained nuclear DNA).

Fluorescence Cell Sort: Hep-2 cells were grown to about 80% confluency in T25 Cell culture flask and were co-infected with HSV-1 at a MOI of 1.0 for 24 h along with the indicated inhibitor compounds and control. After trypsinization, the cells were fixed in 4% paraformaldehyde-PBS. The cells were treated in the same method as described in the immunofluorescence and confocal microscopy method section. The labeled cells were counted in a Nexcelom Vision Cellometer equipped with Brightfield & 2 Fluorescence Channels Filter Set 101: Excitation/Emission Peak: 475 nm/535 nm and Filter Set 202: Excitation/Emission Peak: 525 nm /595 nm. The stained cell samples (20 μl) were pipetted into a Nexcelom counting chamber and inserted into the image cytometer. Bright-field and fluorescent images were then captured and analyzed at four different locations by the Cellometer image analysis software to directly determine the cell concentration of fluorescently labelled cells. The mean ± standard error is reported from three replicates.

### Surface plasmon resonance (SPR) assay

To obtain *in vitro* confirmation of the binding of the isolated compounds to the purified RACK1 protein, the small compound SD-29 and its structural analog SD29-12 were evaluated for their binding potential with the recombinant RACK1A proteins using the Surface Plasmon Resonance (SPR) chip. SPR experiments were carried out with a Biacore T200 equipped with a CM5 sensor chip. Briefly, recombinant RACK1A cysteine-tagged protein was immobilized on flow cell (FC) 2 in HEPES buffered saline (10 mM Hepes, pH 7.4, and 150 mM NaCl, 3 mM CaCl2) using a thiol-coupling kit according to the manufacturer’s protocol, resulting in immobilization levels of 4580 response units (RU). FC1 was only activated and inactivated and used as a reference. SD-29 stock solution was diluted to a final concentration of 100, 75, 50, 25, 10 μM and 1 μM injected in 10 mM Hepes, 150 mM NaCl, 3 mM CaCl2, 1% DMSO, and 0.5% P20. Each injection was repeated three times for 60 s. FC1 signals were deducted from FC2 for background noise elimination. KD_50_ is calculated by the equation: KD_50_ = kd/ka; kd – dissociation rate constant, ka – association rate constant.

### Compound screening

Structure-based screening of two million commercially available diverse compounds was used to screen for small molecules against the RACK1 Y248 phosphorylation site. The diverse set of compounds was preselected in terms of the molecular and topological properties of the RACK1 Y248 binding site. Generation of Receptor Grids for Docking: Grids were generated using Schrodinger’s Glide module. Grid center points were determined from the centroid of each protein’s cognate ligand. To obtain the centroid, the Cartesian coordinates for each atom in the ligand were extracted and the average for each dimension was taken. To determine the size of the grids, a trial-and-error approach to determine the smallest grid size that would allow for the re-docking of all reference ligands was undertaken. The largest reference ligand as our upper size limit was chosen and a grid size of 20 Å on each side was the minimum to allow for it to dock was found. Thus, the grid size for docking simulations was set at 20 Å. The *in-silico* screened compounds were rank ordered based on Glide XP scoring function and top 22 compounds were tested *in-vitro*. Initially, the compounds were screened on our own crystalized plant structure (PDB: 3DM0) [26], and later human RACK1 crystal structure was deduced by other group (PDB: 4AOW). Strikingly, sequence and structure of the Y248 phosphorylation site and K273 sumoylation sites are 100% identical.

## CONCLUSIONS

Viral diseases have historically threatened humanity as well as all other living organisms. The ease with which people travel across continents, urbanization, demographic trends etc. all have accelerated both the emergence and dissemination of viruses around the world. In the area of anti-viral drugs, only few drugs targeting a handful of pathogenic viruses are known to date and more targeted development of antivirals, in the face of emerging viral diseases, are urgently needed. Because viruses have small genome and they mostly use host factors to replicate, it is difficult to find a target to design safe and effective antiviral drugs. Moreover, rapid multiplication of viruses poses a significant barrier to develop durable anti-viral as viruses change over time under selection pressure to become resistance to drugs. Targeting host factors essential for virus replication is an attractive target, however, it is very difficult to find a host factor whose inactivation would not harm the host organism’s cells. In this regard, host factor RACK1 protein which is reported to aid translation of IRES based viral mRNAs, has been established as an attractive target to develop antiviral drugs.

Previously it was shown that cultured cells and whole organism like fruit-fly survived without much adverse effects from RACK1 depletion- indicating that RACK1 is not needed for general translation [3]. Here we have developed host factor RACK1 inhibitor compounds targeting an experimentally validated functional site of the protein and the compounds show promising anti-viral effect in cultured cells. As the host factors are not subject to rapid sequence changes, it is expected that these compounds will be established as durable anti-viral drugs and the drugs are expected not to have detrimental side effects. However, testing in animal models and preclinical trials will be essential to establish the efficacy and to find any potential side effects of these compounds. As the target for drug development is established in this research, it will serve as a guide in the future to develop additional analogs to offer an extended range of drugs for diverse RACK1 mediated disease and developmental pathways. In addition, RACK1 protein is reported to mediate diverse signaling pathways both in plants and in human like drought, salt stress in crops [26, 27] and cancer metastasis in human [57]. The availability of the inhibitor compounds will provide an alternative opportunity to target those pathways as well.
